# Microstructure evolution in a precipitation-hardened high-entropy alloy fabricated by additive manufacturing

**DOI:** 10.1063/4.0001040

**Published:** 2025-10-27

**Authors:** Matthew J Luebbe, Fan Zhang, Haiming Wen

**Affiliations:** 1 NIST, Gaithersburg, MD, USA; 2 Missouri University of Science and Technology, Rolla, MO, USA

## Abstract

High-entropy alloys (HEAs) are a new class of alloys with great potential for harsh environment applications. A precipitation-hardened HEA, (Fe_0.3_Ni_0.3_Mn_0.3_Cr_0.1_)_88_Ti_4_Al_8_, was developed and produced via conventional manufacturing with high strength but low ductility, due to an extensive network of brittle intermetallics that formed after prolonged artificial aging. Additive manufacturing (AM) can often drastically change the microstructure and properties of alloys, so this HEA was also printed using laser powder bed fusion. To induce precipitation and study its kinetics, the printed alloy was subjected to artificial aging at three different temperatures. The aging process was observed in-situ under synchrotron XRD and characterized after aging by SEM, EBSD, and TEM. Results indicated that the as-printed alloy exhibited excellent tensile properties due to its fine grain size and precipitation during printing. Aging induced additional precipitation, especially of the ordered L1_2_ phase. This additional precipitation occurred more quickly than aging precipitation in the conventional manufactured sample, and some precipitates showed a preference for grain boundary nucleation.

